# Clustering of health risk behaviors among school-going adolescents in Mymensingh district, Bangladesh

**DOI:** 10.1186/s12889-023-16766-6

**Published:** 2023-09-23

**Authors:** Lakshmi Rani Kundu, Abdullah Al Masud, Zohurul Islam, Jamil Hossain

**Affiliations:** 1 Department of Public Health and Informatics, Jahangirnagar University, Savar, Dhaka 1342 Bangladesh; 2 Department of Public Health, Hamdard University Bangladesh, Gazaria, Munshiganj 1510 Bangladesh

**Keywords:** Adolescents, Clustering, Health-risk behavior, School-going students, Bangladesh

## Abstract

**Background:**

Adolescents frequently engage in risky behaviors that negatively influence their health and pose a serious public health concern. This study aimed to examine the clustering pattern of health risk behaviors among school-going adolescents in Bangladesh.

**Methods:**

A cross-sectional study was conducted from 15 April to 27 June 2022 among 412 school-going adolescents in Mymensingh district of Bangladesh through a convenience sampling technique. Data were collected via face-to-face interviews using a modified Global School-Based Student Health Survey (GSHS) 2021 questionnaire. Cluster membership was identified using the k-means clustering algorithm. The chi-square test was adopted to explore the association between sociodemographic variables and each cluster membership. The ordinal logistic regression model was employed to examine the predictors associated with cluster membership.

**Results:**

Most of the respondents were female (55.3%) and belonged to the 16-19 years (74.5%) age group. Three behavioral clusters were identified, including Cluster 1: Low-risk taker (50.2%), Cluster 2: Moderate risk taker (39.6%), and Cluster 3: High-risk taker (10.2%). Influential factors of high-risk behavior among adolescents were significantly associated with their age (*p* = 0.03), father’s education who were illiterate (*p* = 0.02), monthly family income >30000 BDT (*p* = 0.04), parent’s those were understanding their child’s problems in most of the time (*p* = 0.001).

**Conclusions:**

The study found that high-risk behaviors are significantly higher among late adolescents, those whose fathers are illiterate, whose monthly income is higher, those whose parents or guardians never realize their child's complications and worries, and those whose parents or guardians never recognize what they did in their leisure time. These findings will help to develop intervention programs, policies, strategies, and curricula in school by the experts following the necessity to adopt the adolescent toward healthy behavior and help to reduce the prevalence of health risk behavior.

## Introduction

Adolescence is a crucial stage for developing risky behaviors that affect adult health [1]. According to UNICEF, 1.3 billion adolescents worldwide account for 16% of the total population [2], and 32 million adolescents (10 to 19) in Bangladesh constitute 21% of the total population [3]. World Health Organization (WHO) estimates that adolescence accounts for about 35% of the worldwide disease burden [4]. Although adolescents are generally considered to be in good health, morbidity and mortality may occur during this time, frequently due to health risk behaviors (HRB) [5]. During adolescence, HRB may intensify low educational attainment, have adverse social and health consequences, and form an unhealthy or socially challenging lifestyle [6]. HRB such as unhealthy dietary behaviors, smoking, excessive smartphone and internet use, insufficient physical activities, and injury or violence-related behavior are common among school-aged adolescents [7]. Therefore, HRB in adolescence is a significant public health concern.

The burden of illness and mortality in adolescence is heavily influenced by substance use, injury-causing behavior, improper personal cleanliness, poor food, and low levels of physical exercise [8, 9]. The clustering of these and other types of HRB may exacerbate low educational achievement and inadequate social and health consequences throughout adolescence and help to create an unfit or socially unsatisfactory lifestyle in adulthood [6]. In this rapid demographic, epidemiological, and nutritional transition period, adolescents face a complicated burden of malnutrition worldwide [10, 11]. Adolescents in Bangladesh have an extremely high prevalence of malnutrition, nutritional deficiencies, overweight, and obesity [12].

The likelihood of dying from non-communicable diseases (NCDs) is raised such as cardiovascular diseases, diabetes, malignancies, and respiratory problems associated with harmful alcohol consumption, poor food, cigarette use, and inactivity [13]. Adolescents are observed to exhibit these behaviors in clusters [14, 15], which may increase their risk of NCDs. A contemporary survey of 2540 school teenagers in Seychelles revealed a high number of people who didn't eat enough fruit and vegetables (61%), didn't get enough exercise (83%), drank a lot of soft drinks (68%) & alcohol (48%), and currently smoked cigarettes (23%) in this population, 81% had 3 or more NCD-related dangerous behaviors [16]. In Bangladesh, a study on adolescent school students of class eight to class ten found NCD-related risk behavior is much that 22.2% consumed less fruit and 47.3% took carbonated soft drinks more than one time daily during the past week, more than two of ten respondents were physical in action along the week [17]. A study reported that adolescents who participate in high-risk behaviors are more likely than their peers who lead healthy lifestyles to suffer from poor health and impaired well-being as adults [18].

The best way to modify preventative programs to serve various subgroups might be determined by understanding how adolescents' behaviors tend to cluster among people in an educational context. Early intervention is therefore essential to prevent high-risk behavior among adolescents since it will enhance population health and diminish the prevalence of many illnesses [19].

Policymakers must know which adolescent groups exhibit high-risk behaviors to ensure this intervention has the desired effect. Methods like cluster analysis and Latent Class Analysis (LCA), which divide individuals into mutually exclusive groups with comparable traits, have been widely employed [20]. Therefore, this study aimed to examine the clustering pattern of health risk behaviors among school-going adolescents in Bangladesh.

## Methods

### Study design and participants

A cross-sectional study was carried out between 15 April and 27 June 2022 among adolescent students in four high schools and three colleges which were selected conveniently in Mymensingh district, Bangladesh. Participants of this study were 13 to 19 years school and college-going students. To participate in the study, subjects had to meet the following criteria: i) students must be from class 8 to 12, ii) age from 13 to 19 years, iii) students who were present in the class at the data collection period, iv) who’re approved to partake in the study by guardians, and v) students who were studying at the co-education institute and Bangla medium. The exclusion criteria included: i) students below class eight and above class 12, ii) ages under 13 and above 19 years, and iii) those who were absent during the data collection. A non-probability sampling (convenience sampling) method was employed to recruit the study participants.

### Sample size determination

The following formula was adopted for the determination of sample size:

$$n=\frac{{z}^{2}pq}{{d}^{2}}=\frac{{\left(1.96\right)}^{2}\times 0.5\times 0.5}{{\left(0.05\right)}^{2}}\approx 384$$

where *z* = 1.96 at 5% level of significance

*d* = 5% acceptable margin of error (d =0 .05)

*p* = sample proportion assumed as 0.5 since this value provides the maximum sample size.

*q* = 1-p = 0.5

Hence, the minimum required sample size is 384. However, 412 participants were recruited to assure the strength of the study.

### Data collection tools and procedure

Data were collected via face-to-face interviews through a modified questionnaire, the Global School-Based Student Health Survey (GSHS) 2021 core questionnaire modules [21]. The questionnaire consists of 8 modules. The first module comprised socio-demographic related questions, six modules included dietary behavior, hygiene behavior, smartphone and internet use, tobacco and tobacco product use, physical activity, and injury or violence-related behavior, and the last module consists of protective factors-related questions. The questionnaire was primarily prepared in English and then transferred into the regional language (Bengali). In this study, Bangla version of the questionnaire was utilized to collect data from the participants. Four enumerators were employed for data collection. The enumerators were provided 5 days of day-long offline training by one of the faculty members of the Department of Public Health and Informatics, Jahangirnagar University, Savar, Dhaka-1342. We invited 435 participants, out of them 23 declined participation during data collection. Therefore, a total of 412 participated in the study, resulting in a 94.71% response rate.

## Measures

### Socio-demographic measure

The socio-demographic characteristics of the respondents were collected using closed-ended questions including their gender, age, educational level, residence, monthly family income, father’s education, and mother’s education.

### Dietary behavior

Daily fruit intake was measured by querying the respondents how often they typically consumed fruits in the previous 7 days (response choices included ‘did not eat fruits’ to ‘4 or more times per day’). Their response was re-categorized to “no consumption”, “1-2 times”, and 3 times or more per day”.

In addition, participants were questioned about how many days in the previous week they had consumed vegetables. We re-categorized the answer options to “no consumption”, “1-2 times”, and 3 times or more per day”.

### Hygiene-related behavior

Oral health was evaluated by querying respondents how frequently they had brushed their teeth in the previous 30 days (response choices included “did not brush or clean teeth” to “3 times or more per day”. We re-categorized the response to “sometimes” if the answer was ‘did not brush or clean teeth’ or ‘did occasionally’, “1 time daily”, and “2 times or more” if the response was ‘2 times’ or ‘3 or more times daily’.

Additionally, respondents were questioned regarding how frequently they had cleaned their hands after using the washroom over the previous 30 days (response choices ranging from “never” to “always”). Their response was re-categorized to “Poor” if the answer was ‘never’ or ‘rarely’, “Sometimes” if the answer was ‘occasionally’, and “Adequate” if the answer was ‘most of the time’ or ‘always’.

### Smartphone and internet use

Participants were asked how many hours a day they used a smartphone and the internet (answer options were “less than 1 hour” to “more than 6 hours”). Their response was re-categorized to “less than 1 hour”, “1 to 2 hours” and “3 or more hours”.

### Tobacco and tobacco product use

Tobacco use was evaluated by asking respondents if they had ever tried cigarette smoking. They were also asked their age when they first attempted smoking a cigarette (answers were “Never smoked”, “7 years old or younger” to “18 years old or older”). Their response was re-categorized to “Never smoked”, “13 years or less” and “14 years and above”.

In addition, participants were questioned about how many days they had smoked cigarettes and smokeless tobacco products during the past 30 days (answers were categorized as “0 days”, 1-9 days”, and “≥10 days”.

### Physical activity

Participants were questioned on how many of the previous seven days they had engaged in at least 60 minutes of physical activity each day. They were also queried how many days in the last seven days they had walked or cycled to school or returned from school. For simpler interpretation of the findings, their answers were divided into “0 days”, “1-4 days”, and “5-7 days”.

### Injury or violence-related behavior

Respondents were questioned how many times they had been severely injured in the last 12 months (answer choices ranged from ‘0 times’ to ‘12 or more times’) and the data were categorized into 3 levels “Never”, “Once” and “More than once” in the analysis, for easier interpretation of the findings. They were also asked how many times they had physically attacked (responses were categorized as “0 times”, “1 or 3 times”, and “4 or more times”).

### Protective factors

Respondents were questioned about how many days they had missed classes or school without permission in the past 30 days (responses were divided into “0 days”, 1-5 days, and 6 or more days”). They also asked how often they were able to talk to someone about difficult complications and worries, and how often their parents or guardians recognize their difficulties and worries.

Parental monitoring during the past 30 days was evaluated by asking participants how frequently their parents or guardians knew what they did in their spare time in the previous 30 days. The response choices were “Never”, “Rarely”, “Sometimes”, “Most of the time”, and “Always”. For simpler interpretation of the findings, their responses were re-categorized to “Never” if the answer was ‘never’ or ‘rarely’, “Sometimes” if the answer was ‘sometimes’, and “Most of the time” if the answer was ‘most of the time’ or ‘always’.

## Statistical analysis

Descriptive statistics (frequencies and percentages) were calculated to represent the data. Then cluster analysis was utilized to classify the respondents according to their health-related behaviors. Six modules including dietary behavior, hygiene behavior, smartphone and internet use, tobacco and tobacco product use, physical activity, and injury or violence-related behavior were used for identifying clustering pattern. First, the number of clusters found using agglomerative hierarchical clustering by means of Ward’s method, which uses squared Euclidean distance as a similarity measure. Subsequently, in order to maximize within group similarities, the k-means clustering algorithm was computed that demonstrate the final set of clusters. The chi-square test was adopted to explore the association between sociodemographic variables and each cluster membership. The ordinal logistic regression model was employed to examine the predictors associated with cluster membership. A *p*-value <0.05 was used for statistical significance. All analysis was accomplished by using SPSS software version 26.0.

## Results

### Background characteristics of the respondents

A total of 412 adolescents engaged in the study, the majority of respondents' age group were between 16-19 years (74.5%), and most were female (55.3%) participants. They mainly studied in the Secondary class (57.8%), and 71.6% lived in rural areas. Among the participants, only 16.7% of the participants' fathers and 13.6% of mothers were illiterate. About 62.9% of participants' family monthly incomes were less than 20000 BDT (1 BDT = 0.0093 US$ on 22 May 2023). The details sociodemographic data of the respondents are depicted in Table 1.

**Table 1:** Baseline characteristics of the study participants

**Variables**	**Categories**	**Frequency (*n*)**	**Percentages (%)**
Age	13-15	105	25.5
	16-19	307	74.5
Gender	Female	228	55.3
	Male	184	44.7
Class	Secondary	238	57.8
	Higher Secondary	174	42.2
Residence	Rural	295	71.6
	Urban	117	28.4
Father’s Education	Illiterate	69	16.7
	Primary	105	25.5
	Secondary	116	28.2
	Higher Secondary or above	122	29.6
Mother’s Education	Illiterate	56	13.6
	Primary	154	37.4
	Secondary	126	30.6
	Higher Secondary or above	76	18.4
Monthly family income (BDT)	>30000	42	10.2
	20000-30000	111	26.9
	<20000	259	62.9

*Note: BDT* Bangladeshi Taka, *1 BDT* 0.0092 US$ in 06 July, 2023

### Behavioral characteristics of the respondents

Table 2 illustrates the distribution of dietary, hygiene, lifestyle, violence and protective factors -related behavior of the participants. About 83.7% of the participants consumed fruit 1-2 times per day a week whereas 4.7% did not take any fruit in the last 7 days. Similarly, in the case of vegetable consumption, the majority (82.3%) are eating vegetables 1-2 times per day a week, and 6.1% did not consume any vegetables. Regarding hygiene-related behavior, the majority (63.6%) of adolescents brushed tooth 2 times per day, 86.2% washed their hands before eating, and 82.5% washed their hands after the toilet adequately.

**Table 2:** Distribution of dietary, hygiene, lifestyle, violence and protective factors related behaviors among adolescents

**Components**	**Variables**	**Categories**	**Frequency (*n*)**	**Percentages (%)**
Dietary and hygiene related behaviors	Fruit consumption in the last 7 days	3 times or more per day	20	4.9
		1-2 times per day	345	83.7
		No consumption	47	11.2
	Vegetable consumption in the last 7 days	3 times or more per day	48	11.7
		1-2 times per day	339	82.3
		No consumption	25	6.1
	Tooth brushing in the last 30 days	2 times or more per day	262	63.6
		1 time per day	143	34.7
		sometimes	7	1.7
	Hand washing before eating in the last 30 days	Adequate	355	86.2
		Sometimes	30	7.3
		Poor	27	6.6
	Hand washing after using the toilet	Adequate	340	82.5
		Sometimes	43	10.4
		Poor	29	7.0
Lifestyle-related behaviors	Weekly physical activity at least 1 hour	5-7 days	96	23.3
		1-4 days	183	44.4
		0 days	133	32.3
	Walking or cycling	5-7 days	194	47.1
		1-4 days	76	18.4
		0 days	142	34.5
	Smartphone use time	≥3 hours	52	12.6
		1 to 2 hours	165	40.0
		<1 hour	195	47.3
	Daily internet browsing hour	≥3 hours	55	13.3
		1 to 2 hours	191	46.4
		<1 hour	166	40.3
	Gaming hours	≥3 hours	76	18.4
		1 to 2 hours	263	63.8
		<1 hour	73	17.7
	Daily sleep hours	>8 hours	112	27.2
		7 to 8 hours	64	15.5
		<7 hours	236	57.3
	Smoking cigarettes	No	383	93.0
		Yes	29	7.0
	Age of first smoking tried	Never smoked	383	93.0
		≤13 years	9	2.2
		≥14 years	20	4.9
	Smoked during the past 30 days	0 days	388	94.2
		1-9 days	11	2.7
		≥10 days	13	3.2
	Use of smokeless tobacco products in the last 30 days	0 days	365	88.6
		1-9 days	39	9.5
		≥10 days	8	1.9
Violence and protective factors related behavior	Times of serious injury	Never	253	61.4
		Once	95	23.1
		More than once	64	15.5
	Times were physically attacked	0 times	334	81.1
		1 or 3 times	66	16.0
		4 or more times	12	2.9
	Bullying at school	No	383	93.0
		Yes	29	7.0
	Class missing without permission	0 days	214	51.9
		1-5 days	169	41.0
		6 or more days	29	7.0
	School student’s kind and helpfulness	Most of the time	222	53.9
		Sometimes	106	25.7
		Never	84	20.4
	Able to talk about difficulties or worries	Most of the time	107	26.0
		Sometimes	162	39.3
		Never	143	34.7
	Parent’s understanding of problems	Most of the time	143	34.7
		Sometimes	133	32.3
		Never	136	33.0
	Parental monitoring during the past 30 days	Most of the time	148	35.9
		Sometimes	130	31.6
		Never	134	32.5

Among the participants, 32.3% had no physical activities in a week, and 44.4% of participants had 1-4 days of physical activity in a week, but more than 65% had good walking or cycling habits. The study has shown that 12.6% used the smartphone for 3 or more hours, 13.3% browsed the internet for 3 or more hours, and 17.7% played games for less than 1 hour per day.

About 57.3% of the respondents reported sleeping less than 7 hours daily. The majority (93%) were not smoked cigarettes. Only 7% were smoking, mostly starting cigarettes after 13 years old, and 3.2% smoked cigarettes more than 10 days per month. The study results also revealed that only a few participants used smokeless tobacco more than 10 days a month.

In this study, almost 16% had two or more severe injuries, only 3% were physically involved more than 4 times, and 7% were bullied in school. Nearly half (51.9%) of the adolescents never missed class, 41% missed one to five days of school without permission, and 20% never felt kind and supportive during the past 30 days. Regarding their parents’ perspective, more than one-third (34.7%) of the respondents' parents knew about their child's problem, whereas 33% never understood the problems of their child’s, and 32.5% never monitored what their child’s doing in their free time (Table 2).

### Cluster groups

**Fig. 1:**
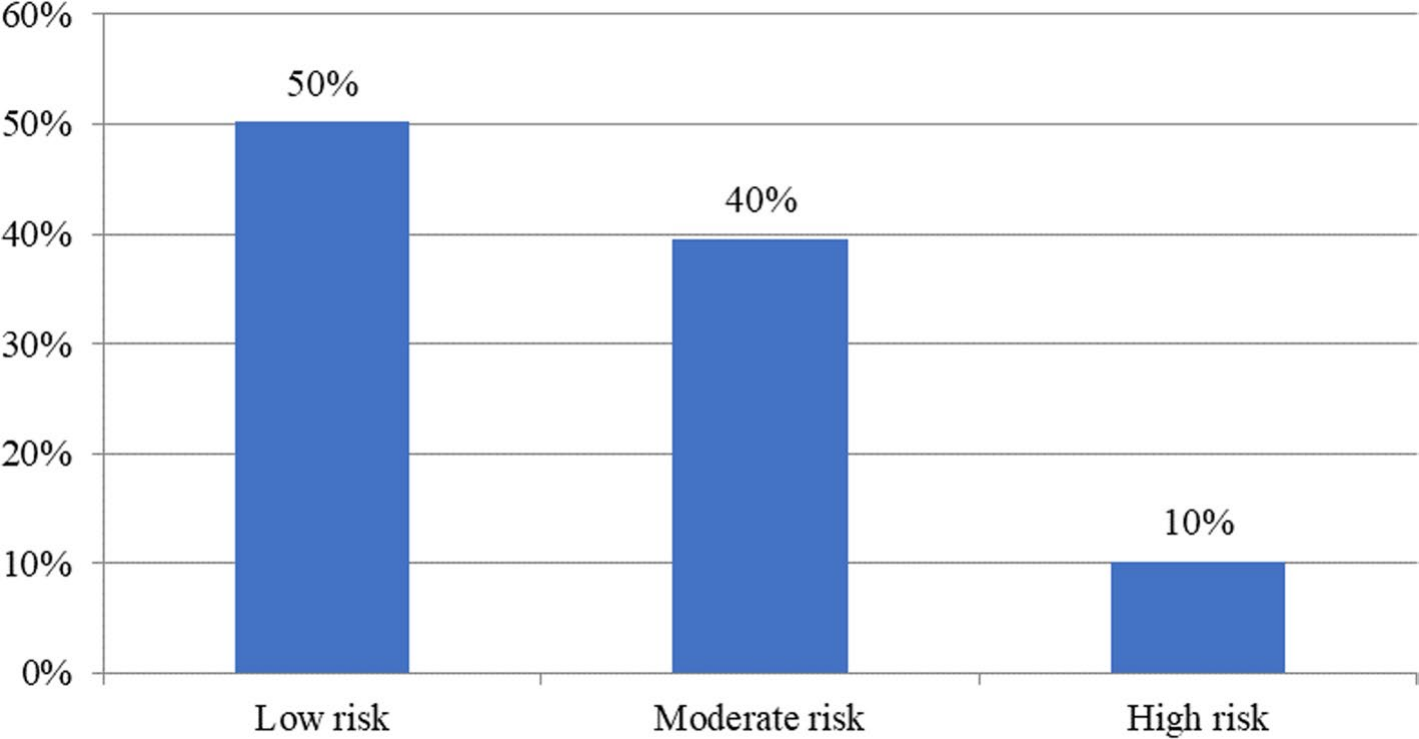
Distribution of health risk behaviors cluster

On the basis of health behaviors, three distinct clusters were identified. The bar diagram (Fig. 1) depicts the distribution of three clusters: Cluster 1 (low-risk taker), Cluster 2 (moderate-risk taker), and Cluster 3 (high-risk taker). We found that 50.2%, 39.6%, and 10.2% of the respondents were located in Cluster 1, Cluster 2, and Cluster 3, respectively.

### Behavioral characteristics of the clusters

**Fig. 2:**
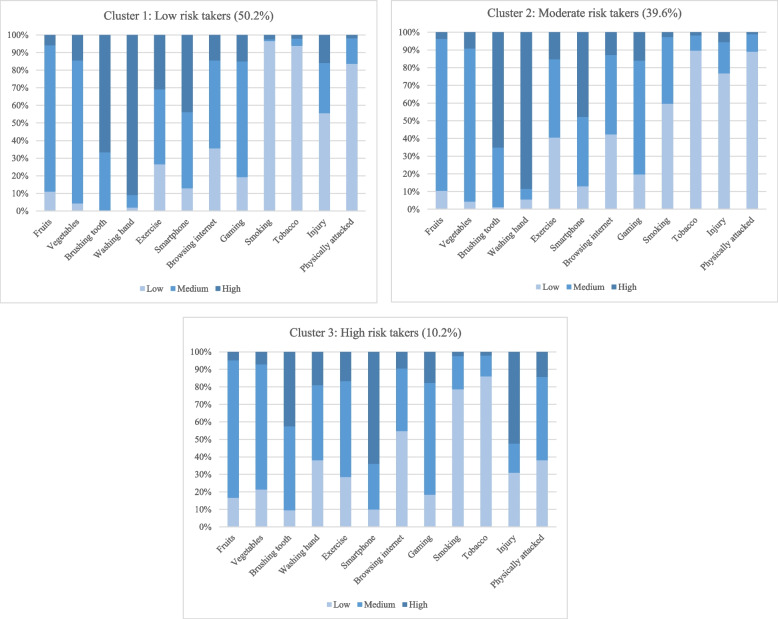
Stacked bar diagrams showing the distribution of health risk behaviors in each cluster

Findings on the behavioral characteristics of each cluster are portrayed in Fig. 2.

*Cluster 1 (low risk-takers)* was characterized by adolescents who reported the highest daily fruit and vegetables consumption, abstinence from tobacco, and much better hygiene maintains (hand washing and tooth brushing) compared to other cluster members. They reported least involved in injuries and acts of violence than did adolescents in cluster 3, and adolescents in cluster 1 reported highest prevalence of physically active (31% reported at least an hour every 5-7 days). Additionally, the lowest percentage of adolescents spent more time in smartphone, internet use and gaming compared to cluster 2 and cluster 3.

*Cluster 2 (moderate risk-takers)* comprised adolescents who reported very low physically attacked (1% stated 4 or more times in the last year), and had the least reports of serious injury (6%). In addition, they spent less time in smartphone, internet use and gaming as well as their oral hygiene and washing habit were better than that of adolescents in cluster 3. However, just 4% of adolescents in cluster 2 stated eating fruits three or more times per day, second lowest reported consume vegetables three or more times per week, and physically active five or more days per week.

*Cluster 3 (high risk-takers)* was characterized by adolescents who stated the most involvement in injury and violence (52%), the worst oral hygiene and handwashing habits, lowest prevalence of physically active, and the highest reports of smartphone use (64%). Furthermore, least reported the daily consumption of fruit and vegetables three or more times per week, and highest involvement in physically attacked (18%) in cluster 3.

### Socio-demographic characteristics of behavioral clusters

The socio-demographic characteristics of the respondents in each cluster are depicted in Table 3. Results revealed no significant difference in age, class, residence, mother's education, father’s education, and monthly family income across the clusters. In this study, we found that the maximum of high-risk takers (Cluster 3) were aged between 16-19 years (66.7%) and were male respondents (73.8%). A statistically significant relationship existed between gender and cluster membership (*p* < 0.001). A higher proportion of secondary school adolescents (62%) belong to high-risk takers (Cluster 3) as opposed to adolescents who study higher secondary. The majority (73.8%) of high-risk takers (Cluster 3) came from rural areas. In Cluster 3 (High risk-takers), there were more teenagers whose parent’s never understanding about problems (50.0%; *p* = 0.002) and adolescents those parents never monitored their activities in their free time (45.2%; *p* = 0.002) compared to Clusters 1 and 2.

**Table 3:** Socio-demographic characteristics and protective factors of the 3 behavioral clusters

**Variables**	**Categories**	**Cluster 1** **(Low-risk takers)** ***n* (%)**	**Cluster 2 (Moderate** **risk takers)** ***n* (%)**	**Cluster 3 (High-risk takers)** ***n* (%)**	***p*-value**
Age	13-15	48 (23.2)	43 (26.4)	14 (33.3)	0.367
	16-19	159 (76.8)	120 (73.6)	28 (66.7)	
Gender	Female	116 (56.0)	101 (62.0)	11 (26.2)	**<0.001**
	Male	91 (44.0)	62 (38.0)	31 (73.8)	
Class	Secondary	121 (58.5)	91 (55.8)	26 (61.9)	0.746
	Higher Secondary	86 (41.5)	72 (44.2)	16 (38.1)	
Residence	Rural	144 (69.6)	120 (73.6)	31 (73.8)	0.654
	Urban	63 (30.4)	43 (26.4)	11 (26.2)	
Father’s education	Illiterate	31 (15.0)	28 (17.2)	10 (23.8)	0.389
	Primary	57 (27.5)	39 (23.9)	9 (21.4)	
	Secondary	52 (25.1)	49 (30.1)	15 (35.7)	
	Higher Secondary or above	67 (32.4)	47 (28.8)	8 (19.0)	
Mother’s education	Illiterate	30 (14.5)	20 (12.3)	6 (14.3)	0.296
	Primary	75 (36.2)	62 (38.0)	17 (40.5)	
	Secondary	63 (30.4)	46 (28.2)	17 (40.5)	
	Higher Secondary or above	39 (18.3)	36 (21.4)	2 (4.8)	
Monthly family income (BDT)	>30000	16 (7.7)	22 (13.5)	4 (9.5)	0.244
	20000-30000	51 (24.6)	48 (29.4)	12 (28.6)	
	<20000	140 (67.6)	93 (57.1)	26 (61.9)	
Able to talk about difficulties or worries	Most of the time	64 (30.9)	40 (24.5)	3 (7.1)	**0.006**
	Sometimes	74 (35.7)	62 (38.0)	26 (61.9)	
	Never	69 (33.3)	61 (37.4)	13 (31.0)	
Parent’s understanding of problems	Most of the time	85 (41.1)	53 (32.5)	5 (11.9)	**0.002**
	Sometimes	55 (26.6)	62 (38.0)	16 (38.1)	
	Never	67 (32.4)	48 (39.4)	21 (50.0)	
Parental monitoring	Most of the time	92 (44.4)	47 (28.8)	9 (21.4)	**0.002**
	Sometimes	63 (30.4)	53 (32.5)	14 (33.3)	
	Never	52 (25.1)	63 (38.7)	19 (45.2)	

*Note: BDT* Bangladeshi Taka, *1 BDT* 0.0092 US$ in 06 July, 2023

### Factors associated with behavioral cluster membership

In Table 4, the findings of the multivariable ordinal logistic regression model revealed that cluster membership was significantly associated with age, fathers' education, monthly family income, and parents' understanding of their child's problems. Male adolescents were 1.19 times more likely to have high-risk behaviors (AOR: 1.19; 95% CI: 0.81-1.76) compared to female adolescents.

**Table 4:** Ordinal logistic regression model of factors influencing high-risk behaviors among adolescents

**Variables**	**Categories**	**COR (95% CI)**	***p*-value**	**AOR (95% CI)**	***p*-value**
Gender	Male	1.31 (0.90, 1.91)	0.153	1.19 (0.81, 1.76)	0.368
	Female	Reference		Reference	
Age	16-19	1.32 (0.87, 2.02)	0.028	1.65 (1.05, 2.60)	**0.030**
	13-15	Reference		Reference	
Residence	Rural	1.21 (0.80, 1.83)	0.371	1.16 (0.74, 1.81)	0.524
	Urban	Reference		Reference	
Father’s Education	Illiterate	1.62 (0.92, 2.86)	0.017	2.54 (1.15, 5.59)	**0.021**
	Primary	1.06 (0.64, 1.76)	0.829	1.20 (0.64, 2.26)	0.577
	Secondary	1.57 (0.96, 2.57)	0.072	1.68 (0.95, 2.96)	0.075
	Higher Secondary or above	Reference		Reference	
Mother’s Education	Illiterate	1.06 (0.54, 2.08)	0.855	0.66 (0.27, 1.63)	0.367
	Primary	1.26 (0.74, 2.15)	0.392	1.24 (0.64, 2.41)	0.527
	Secondary	1.27 (0.73, 2.20)	0.400	1.18 (0.63, 2.18)	0.604
	Higher Secondary or above	Reference		Reference	
Monthly family Income (BDT)	>30000	1.65 (0.89, 3.06)	0.048	2.03 (1.03, 3.99)	**0.041**
	20000-30000	1.33 (0.87, 2.04)	0.192	1.41 (0.91, 2.20)	0.125
	<20000	Reference		Reference	
Able to talk about difficulties or worries	Most of the time	0.60 (0.37, 0.99)	0.047	0.74 (0.44, 1.23)	0.243
	Sometimes	1.25 (0.81, 1.92)	0.311	1.33 (0.81, 2.16)	0.259
	Never	Reference		Reference	
Parent’s understanding of problems	Most of the time	0.57 (0.36, 0.89)	0.016	0.39 (0.22, 0.68)	**0.001**
	Sometimes	1.21 (0.76, 1.90)	0.421	0.67 (0.41, 1.09)	0.109
	Never	Reference		Reference	
Parental monitoring	Most of the time	0.39 (0.24, 0.62)	<0.001	0.57 (0.26, 0.82)	**0.005**
	Sometimes	0.69 (0.43, 1.09)	0.109	0.48 (0.24, 0.73)	0.246
	Never	Reference		Reference	

*Note: BDT* Bangladeshi Taka, *1 BDT* 0.0092 US$ in 06 July, 2023

The likelihood of being a member of a greater risk-taking cluster rises with teenage age (AOR: 1.65; 95% CI: 1.05-2.60). Rural adolescents were 1.16 times more likely to belong to cluster 3 (high-risk behavior) (AOR: 1.16; 95% CI: 0.74-1.81) than urban adolescents. Adolescents those fathers who were illiterate were 2.54 times more likely to have high-risk behavior (AOR: 2.54; 95% CI: 1.15-5.59) compared to those fathers who were higher educated.

In contrast, we found a negative association in mothers with illiterate education, 34% less likely to develop high-risk behaviors (AOR: 0.66; 95% CI: 0.27-1.63) as opposed to those adolescents’ mothers who had higher education. Adolescents who could talk to someone about difficult complications and worries most of the time past 30 days were 26% less likely to have high-risk behavior (AOR: 0.74; 95% CI: 0.44-1.23) as opposed to those who never shared their problems. Again, those parents or guardians who recognize their child's complications and worries most of the time were almost 60% less likely to belong to cluster 3 (AOR: 0. 0.39; 95% CI: 0.22-0.68) compared to those parents who never understand their child's problems and worries. Parental monitoring was also linked to cluster membership, as it was less likely for adolescents whose parents or guardians always realize what they worked in their leisure time (AOR: 0.57; 95% CI: 0.26- 0.82) than for those whose parents or guardians never realize what they worked in their leisure time to belong to one of the higher risk clusters (i.e., moderate or high risk-takers) in comparison to Cluster 1.

## Discussion

This study highlights the clustering pattern of health risk behaviors among school-going adolescents living in the Mymensingh district of Bangladesh. The current study evaluated high-risk behaviors such as dietary, hygiene, tobacco use, smartphone and internet use, physical activity, and injuries or violence-related behavior. An earlier study identified the clustering pattern of specific high-risk behavior among Bangladeshi adolescents [22], whereas our research focused on the level of risk. According to our study, tobacco use was the least frequent behavior among Bangladeshi teenagers, whereas physical inactivity was the most frequent. This result is similar to the study carried out by Cheah et al. (2019), who observed that physical activity was the most common behavior and alcohol consumption was the least among Malaysian adolescents [19]. This study revealed that among the teenagers who are residing in Mymensingh, Bangladesh, there is a clustering of injuries, tobacco use, smartphone or internet use, poor cleanliness, inadequate physical activity, and eating habits. We identified three health risk behavior clusters. Our study's result is consistent with the study conducted in Kenya [14]. Four behavioral groups were produced by three research on HRB clustering among teenagers from the Netherlands [23], China [24], and the USA [25]. On the other hand, one study conducted in London identified two behavioral clusters [15]. The variety in the behavioral parameters used for clustering and the discrepancy in contextual factors may be the causes of the discrepancy in the number of clusters produced.

These findings revealed that high-risk behavior was higher among male adolescents than female adolescents, and most high-risk takers (Cluster 3) were late adolescents. Similar to our results, older age and being male were linked to belonging to riskier behavioral clusters among Chinese teenagers [26], Bahamas adolescents [27], adolescents in Kenya [14], and Malaysia adolescents [19], while neither of these characteristics was linked to belonging to a cluster among Dutch adolescents [23]. Adolescents who could never share their difficulties and worries with someone were at higher odds of belonging to high-risk takers (Cluster 3). Furthermore, adolescents those parents or guardians never realized their complications and worries were at higher odds of belonging to Cluster 3 (high-risk behavior) compared to Cluster 1 (low-risk behavior) and Cluster 2 (moderate-risk behavior).

We observed that parental monitoring was significantly associated with cluster membership. The study results revealed that adolescents whose parents or guardians never monitored their free time and what they did were higher odds of belonging to high-risk behavior. This result highlights the crucial and immediate need for a thorough investigation into parenting behavior and its underlying causes to pinpoint particular obstacles and possibilities for encouraging helpful parenting behavior among adolescent caregivers in Bangladesh. This finding is similar to another study conducted among Kenyan adolescents [14]. According to further research, parental monitoring promotes strength, moderates peer-influenced risk behavior, and has long-lasting impacts on children's behavior that last until late adolescence [28]. This result indicates that parental monitoring interventions might help Bangladeshi adolescents who suffer from HRB. Several possible elements of such interventions include promoting communication between parents and children, increasing parental involvement in teenagers' activities including activities in school, and instruction in social skills to teenagers [29, 30]. However, these intervention elements might be necessary for testing and adaptation to the Bangladeshi setting.

## Strengths and limitations

So far, this the first study that clustered the health risk behavior among Bangladeshi adolescents. However, this study has some limitations. Firstly, this was a cross-sectional study conducted on school days, so the student absent on that day is not included in the study. Secondly, this study was done in an Upazila (Sub-districts) and 3 institutes situated in the municipal area, so this result may not be generalized to the country and may also be different from rural areas institutes. Thirdly, the study is additionally constrained by the small sample size. The study also limited by convenient sampling. Future studies need to overcome these constraints through longitudinal designs with larger and more representative samples by employing probability sampling.

## Conclusions

This study found the prevalence of health risk behavior and clustering pattern of risk behaviors among school-going adolescents. We identified three behavioral clusters among adolescents. Age, fathers' education, monthly family income, and parents' understanding of their child's problems were significantly associated with adolescents' risky behavior. The likelihood of having high risk is substantially higher among late adolescents, those whose fathers are illiterate, whose monthly income is higher, those whose parents or guardians never realize their child's complications and worries, and those whose parents or guardians never recognize what they worked in their leisure time. These findings would be conducive to developing intervention programs, policies, strategies, and curricula in school by experts following the necessity to adopt the adolescent toward healthy behavior and help mitigate the prevalence of health risk behavior.

## Data Availability

The data that support the findings of this study are available from the corresponding author upon request.

## Abbreviations

HRB: Health risk behavior
NCD: Non-communicable disease
GSHS: Global School-Based Student Health Survey
95% CI: 95% Confidence Interval
COR: Crude odds ratio
AOR: Adjusted odds ratio
